# Navigating Data Acquisition and Data Quality Validation of Large Databases: Practical Lessons

**DOI:** 10.23889/ijpds.v9i5.1657

**Published:** 2024-09-18

**Authors:** Nedeene Hudema, Amanda Hutton, Nirmal Sidhu, Xinya Lu, Xue Feng

**Affiliations:** 1 Saskatchewan Health Quality Council

## Objective

Have you ever been frustrated when working to acquire data? Have you experienced the trials and tribulations of readying data? ‘Best laid plans often go awry’. ‘A problem shared is a problem halved’ and we’d like to share our experiences and lessons learned with you.

We are a Canadian provincial organization with over 20 years experience working with large comprehensive data currently in the midst of validation work on newly acquired data. This behind the scenes and sometimes forgotten work can take considerable time, but it is crucial and integral for research. 

## Approach

Data acquisition is complex, particularly when adding new data to existing data files. Many validation steps are vital to ensuring that data used in research are the highest quality possible and limitations are understood. The process began with preparing the agreement and list of new variables to add in 2018 for 4 databases (hospital, drug, physician, emergency), to signatures in 2023, and it is ongoing. While we have a plan to do this work, it requires flexibility and constant updates based on new information learned. 

## Conclusion

Investigating errors, deciding level of acceptance of errors, managing relationships, and communication are the most critical aspects of the work. Contingency plans are important when it takes longer than expected. Data are never perfect, but a thoughtful approach to validation and documentation of limitations and decisions support using the data appropriately. Also, a kind demeanor, an open mind, and a sense of humour can go a long way!

